# Dementia Caregivers' Preferences for Involvement in Delirium Care During Hospitalization

**DOI:** 10.1111/jgs.70602

**Published:** 2026-08-03

**Authors:** Blair P. Golden, Brooke Johnson, Laura Block, Clark Benson, Kristin Merss, Kellia J. Hansmann, Gina Green‐Harris, Sharon K. Inouye, Andrea Gilmore‐Bykovskyi

**Affiliations:** ^1^ Department of Medicine University of Wisconsin‐Madison School of Medicine and Public Health Madison Wisconsin USA; ^2^ College of Nursing University of Utah Salt Lake City Utah USA; ^3^ BerbeeWalsh Department of Emergency Medicine University of Wisconsin‐Madison School of Medicine and Public Health Madison Wisconsin USA; ^4^ University of Wisconsin – Madison, School of Nursing Madison Wisconsin USA; ^5^ Department of Family Medicine and Community Health University of Wisconsin‐Madison School of Medicine and Public Health Madison Wisconsin USA; ^6^ Center for Community Engagement and Health Partnerships University of Wisconsin‐Madison School of Medicine and Public Health Madison Wisconsin USA; ^7^ Wisconsin Alzheimer's Institute Regional Milwaukee Office Milwaukee Wisconsin USA; ^8^ Harvard Medical School Boston Massachusetts USA; ^9^ Marcus Institute for Aging Research Hebrew SeniorLife Boston Massachusetts USA

**Keywords:** caregiving, delirium, dementia

## Abstract

**Background:**

Nearly half of hospitalized adults with dementia develop delirium superimposed on dementia (DSD), which is associated with adverse outcomes. Family caregivers play an essential role in preventing, identifying, and responding to DSD, yet relatively little is known about how caregivers experience and respond to acute cognitive changes during illness and their preferences for involvement in DSD care.

**Methods:**

We performed a qualitative study of caregivers (*N* = 22) for patients with dementia admitted to two hospitals within a single health system. Because caregivers may be engaged in DSD prevention prior to an episode of DSD, we included caregivers to patients with a history of delirium (*N* = 19) and without (*N* = 3). We conducted semi‐structured interviews during hospitalization or within 1 week of discharge focused on caregiver experiences with acute cognitive changes during illness and preferences for involvement in DSD care. Interviews were analyzed using thematic analysis.

**Results:**

Caregivers readily described experiences identifying acute cognitive changes during illness, but few were familiar with the term “delirium.” Caregivers described navigating acute cognitive changes via common phases of (1) recognizing acute cognitive changes, (2) interpreting their meaning, and (3) deciding how to act. Some recognized and interpreted changes as normative in the context of dementia, while others expressed concern about the potential impact and seriousness of symptoms. Caregivers described several factors—perceived caregiving role and context, care priorities, delirium knowledge, and distress from delirium—that affected how they navigated symptoms as well as their preferences and motivations for involvement in DSD care. Participants reported substantial variation in factors that influenced their involvement in DSD care.

**Conclusions:**

Caregivers navigate and engage with acute cognitive changes during illness in heterogeneous ways, shaped by their preferences for involvement in care. Findings support flexible, caregiver‐centered intervention strategies for DSD that recognize variability in caregiver roles, needs, and readiness for engagement.

## Summary

- Key Points
  ○ While family caregivers play a vital role in prevention, identification, and management of delirium superimposed on dementia (DSD) during hospitalization, the best strategies for involving caregivers in DSD care remain poorly understood, with little attention paid to how caregivers interpret and respond to acute cognitive changes or participate in care.
  ○ Caregivers described navigating acute cognitive changes during illness through three interconnected phases of recognizing changes, interpreting changes, and deciding to act, although there was notable variation in how caregivers navigated these phases.
  ○ Caregivers described highly individual factors (including perceived caregiving role and context, care priorities, delirium knowledge, and distress from delirium) that affected how they navigated symptoms as well as their preferences for involvement in DSD care.
- Why does this paper matter?
  ○ Family caregiver experiences navigating acute cognitive changes and preferences for involvement in DSD care are not uniform. These findings highlight the importance of developing flexible, caregiver‐centered interventions for DSD that can be adapted to account for variability in caregiver roles, needs, and readiness for engagement.

## Graphical abstract

Family caregivers to hospitalized patients with dementia describe highly individual factors that affect how they navigate acute cognitive changes during illness and their preferences for involvement in delirium care.

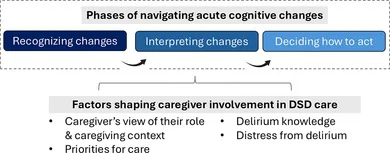

## 1 Introduction

Almost half of all hospitalized adults with dementia develop delirium, a clinical syndrome characterized by acute fluctuations in cognition and attention [1, 2]. Delirium superimposed on dementia (DSD) is associated with adverse health impacts—including prolonged hospitalization, accelerated cognitive and functional decline, and increased mortality—and exacerbates caregiver strain [1, 3, 4, 5]. Distinguishing DSD from baseline dementia is vital because DSD may signal a reversible medical condition requiring prompt evaluation [6, 7, 8]. Given high rates of hospitalization for people living with dementia, timely identification and management of DSD is a critical priority [6, 9, 10].

Family caregivers play an essential role in identification and management of DSD due to their knowledge of a patient's baseline cognition, which is central to assessing acute cognitive changes and tailoring delirium prevention and management strategies [11, 12]. A recent scoping review found that hospital‐based caregiver interventions for DSD have the potential to reduce delirium risk and alleviate caregiver strain [12]. Caregiver involvement is also recommended in international practice guidelines for delirium [13, 14]. However, the best strategies for involving caregivers in DSD care remain poorly understood, with little attention paid to how caregivers interpret and respond to DSD or participate in care during hospitalization [12, 15, 16]. Furthermore, it remains unclear if and how these strategies should be tailored to individual patient and caregiver needs and contexts, which may limit their effectiveness [12].

To address this gap, we sought to examine caregiver experiences and perceptions of acute cognitive changes surrounding hospitalization and preferences for involvement in DSD care. This information will be essential to develop optimal strategies for caregiver interventions to prevent, identify, and treat DSD.

## 2 Methods

We conducted a descriptive qualitative study examining family caregivers' experiences with and perceptions of acute cognitive changes among hospitalized persons with dementia. Because DSD is under‐recognized and caregivers are often engaged in prevention efforts before a formal diagnosis, we included caregivers of patients both with and without chart‐documented delirium to capture the diversity of caregivers engaged in real‐world delirium prevention and intervention efforts [17]. Methods and results are reported in accordance with the COnsolidated criteria for REporting Qualitative Research [18]. The University of Wisconsin‐Madison Institutional Review Board approved this study.

### 2.1 Setting and Participants

We recruited caregivers of hospitalized patients with dementia admitted to 2 hospitals (one academic, one community) within a single system. We identified patients with dementia admitted to general care services through chart review. We reviewed the electronic health record to determine whether a physician or advanced practice provider documented a diagnosis of DSD or a synonymous diagnosis (e.g., acute metabolic encephalopathy) during the index hospitalization and/or prior hospitalizations [19, 20]. We confirmed with a hospital clinician that the patient had involved caregiver(s) able to complete an interview in English. Consistent with our focus on caregiver experiences during hospitalization, we defined involved caregivers as individuals interacting with clinicians during hospitalization and included caregivers of individuals residing in both community and facility settings to capture a broader range of experiences. We obtained verbal consent from caregivers prior to interviews. Participants received a $75 honorarium.

### 2.2 Interview Procedures

Our semi‐structured interview guide (Text S1) asked about experiences with acute changes in cognition leading up to and during hospitalization. Because caregivers described acute cognitive changes across both current and prior illness episodes and because we did not prospectively validate DSD diagnoses, we refer to described symptoms as “acute cognitive changes” throughout to reflect this broader experiential framing and avoid misclassification. We asked for the caregiver's perspective on symptom recognition and interpretation, clinician communication about symptoms, and recommendations to improve DSD care, including caregiver preferences for involvement in care.

Interviews took place between April 2024 and January 2025. Consistent with best practices for recruiting caregivers in acute care settings, we prioritized flexibility and participant choice in interview timing and modality [21]. Caregivers were offered the option to complete interviews during hospitalization or within 7 days of discharge, based on their preference and availability. We conducted interviews in‐person, via Zoom or phone based on participant preference. Authors (B.P.G., B.J.), both of whom have clinical experience working with persons living with dementia and qualitative training, conducted the interviews. Our team has experience adapting interviewing techniques across modalities to ensure rich data collection [22]. Interviews lasted 30–45 min, with similar duration across modalities.

We audio‐recorded, transcribed, and de‐identified interviews. We obtained caregiver demographics and assessed caregiver knowledge of delirium, caregiver burden, and caregiver rating of dementia severity using validated instruments [23, 24, 25]. We used these measures to characterize the sample, but did not directly incorporate these measures into qualitative analysis.

### 2.3 Analysis

We analyzed interviews concurrently with data collection using thematic analysis [26]. Consistent with Braun and Clarke's framework, we began analysis with memoing and reviewing interview data to develop familiarity with the data [26]. We then completed iterative coding of key concepts across all transcripts. All transcripts were independently coded by two authors (B.P.G., B.J.), with discrepancies resolved through consensus discussion. To develop codes into categories and then themes, we reviewed coded data and generated categories and themes during meetings with an interdisciplinary team with advanced qualitative expertise (B.P.G., L.B., C.B., K.M., K.J.H., G.G.‐H., A.G.‐B.). Meetings fostered in‐depth review of the data, codes, and themes, peer debriefing, reflexivity and audit trails, enhancing rigor [27]. To enhance analytic rigor and robustness of findings, we conducted analyses to assess whether thematic findings differed across caregivers with varying levels of prior experience with delirium, including caregivers to patients without a known history of delirium.

## 3 Results

We interviewed 22 caregivers providing care for 21 unique patients (2 caregivers provided care for the same patient). Caregivers were primarily women (73%) and White (91%), with half (50%) reporting professional healthcare experience (Table 1). Most caregivers cared for a patient with moderate or severe dementia and reported high levels of caregiver burden and low delirium knowledge. Three caregivers cared for a patient without a history of delirium.

**TABLE 1:** Caregiver and patient demographics.

Caregiver demographics (*N* = 22)^a^		*N* (%)
Gender	Female	16 (73%)
Race (ethnicity)	White (non‐Hispanic)	20 (91%)
	Black (non‐Hispanic)	2 (9%)
Age (years)	25–49	3 (14%)
	50–74	16 (73%)
	≥ 75	3 (14%)
Relationship with patient	Spouse/significant other	6 (27%)
	Child	14 (64%)
	Other	2 (9%)
Educational attainment	High school or equivalent	1 (5%)
	Some college or college degree	19 (86%)
	Graduate degree	2 (9%)
Professional healthcare experience	Yes	11 (50%)^b^
6‐item Zarit Burden Inventory (range 0–24)	Mean (SD)	14.1 (4.7)
Caregiver Delirium Knowledge Questionnaire (range 0–19)	Mean (SD)	12.5 (2.6)

Patient demographics (*N* = 21)		*N* (%)
Gender	Female	11 (52%)
Age (years)	60–74	3 (14%)
	75–89	15 (71%)
	≥ 90	3 (14%)
Living Situation	Private home	10 (48%)
	Assisted Living Facility	7 (33%)
	Other Facility	4 (19%)
History of delirium	Index hospitalization only	5 (24%)
	Prior hospitalization only	6 (29%)
	Both index and prior hospitalization	7 (33%)
	No history	3 (14%)
Quick Dementia Rating System (QDRS) Dementia Severity	Mild Dementia	3 (14%)
	Moderate Dementia	13 (62%)
	Severe Dementia	5 (24%)
Hospital stay characteristics		
Primary hospital service	Medicine	19 (90%)
	Surgery	2 (10%)

^a^ Interview included 22 caregivers providing care for 21 unique patients (2 caregivers provided care for the same patient).

^b^ Includes experience as a nurse (RN/LPN), advanced practice provider (APP), and nursing assistant. No participants were physicians or had experience working in inpatient medical‐surgical wards.

Overall, we identified variation in how caregivers recognized and described acute cognitive changes, interpreted and made sense of these changes, and decided what they wanted to do as a result. Despite variable experiences, there were common phases through which caregivers navigated identifying and responding to these symptoms. Additionally, caregivers described highly individualized factors that shaped their recognition of symptoms, assessment of whether and if to intervene, and preferences for involvement in DSD care. Thematic findings are illustrated in Figure 1 with representative quotes for the phases of navigating acute cognitive changes and factors shaping caregiver involvement shown in Tables 2 and 3, respectively.

**FIGURE 1:**
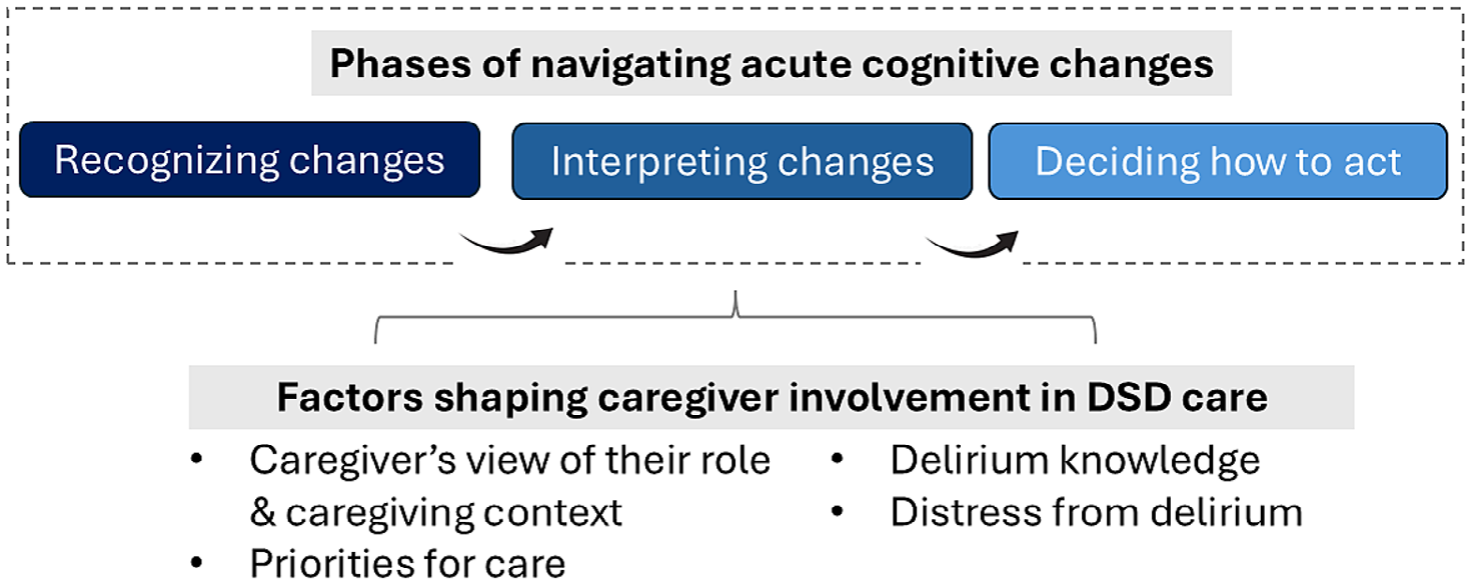
Overview of study findings.

**TABLE 2:** Phases of navigating acute cognitive changes: Themes and representative quotes.

Theme	Representative quote
Recognizing changes	*Not themselves*: “It would be just changes in her behavior. So if, all of a sudden, like typically, because she's a constant mover and talker and actually happy‐go‐lucky, so if, all of a sudden, she became aggressive or slowed way down”—Caregiver 15
	*Not in their right mind*: “She wasn't quite in her right mind, of course. She doesn't know where she is or why she's there.”—Caregiver 14
	*Waxing and waning symptoms*: “Sunday my sister said her day was much better, kind of more of her normal self … but when I came on Monday, she was kind of out of it again. So, she seemed like she had taken steps back.”—Caregiver 13
	*Challenges recognizing changes in dementia*: “I think it would be really tough for a hospital staff for people that don't know these patients. That's the tough thing is, because if you don't know what the baseline is, especially in an Alzheimer's patient”—Caregiver 1
Interpreting changes	*Recalling prior experiences*: “I think a lot of what's going on now is a little bit like deja vu from when she broke her hip.”—Caregiver 14
	*Hypothesizing causes*: “Her condition was created because of the urinary tract infection, which then got into the blood stream. And that type of bacteria in the blood stream, infection, as I understand it, can cause people to have some, even healthy people, if it gets that bad, can be a little delirious” – Caregiver 11
	*Ascribing importance*: “Those are the moments when I'm like, okay, like is she going to like wake up, or is this permanent, or like what's going on, and what should I expect? And even after having seen it, after multiple visits, I still immediately go back to like, okay, like is this her mental state now?”—Caregiver 8
	*Potentially normative with aging*: “It's like that was initially alarming until, you know, we realized that she got better when her infection went away. And it was like, oh, this is just what happens, you know, when you are this age … you have sepsis.”—Caregiver 13
Deciding how to act	*Seeking medical care for work‐up*: “And it was like, no, this man needs help. This is a change in mentation, a change in behavior. It's, there's something going on. He needs to be taken to the ER.”—Caregiver 22
	*Voicing concerns about cognition*: “I know from experience, and actually I'm usually the one to warn them about it that this has been going on”‐ Caregiver 18
	*Relating cognitive baseline*: “And I tend to, because I've been doing this for a while, I tend to offer more baseline right off the bat, so I can't say that they always get a chance to ask me about it.”—Caregiver 16
	*Avoiding potential triggers*: “I do think like she starts developing kind of like that delirium, sending her somewhere else isn't going to make it better. I think it's just going to make it worse.”—Caregiver 6

**TABLE 3:** Factors shaping caregiver involvement in DSD care.

Factor (theme)^a^	Sub‐theme	Representative quotes
Caregiver's view of their role and caregiving context	Advocacy	*Identifies as an advocate*: “I need to make sure advocate for her on what I know, versus what the healthcare system knows.”—Caregiver 1
		Versus *defers to medical expertise*: “Because sometimes I think things are going on, and, you know, as lay people, we don't realize there's any importance to this.”—Caregiver 18
	Proactive planning	*Planning ahead*: “I think it would be beneficial…saying, hey, this is a possibility going down the road, or keep it in your back pocket that they could experience some delirium, you know, either driven from the disease or an infection or something else going on, but looking for those drastic, instant changes, sudden changes in behavior”—Caregiver 15
		Versus *being less proactive*: “But anyways, people like her, I mean, she loves her grandpa, they're very close. But she isn't going to be coming in here and be like, “Let me know,” but she'd be concerned if she was told it could happen.”—Caregiver 5
	Providing expertise regarding patient cognition & picking up on the signs	*Picking up on symptoms that others may not notice*: “I can tell she's not quite herself, but I don't know that anybody else here could tell that.” – Caregiver 6
		Versus *being less likely to detect changes*: “I don't see her a ton. Like it certainly, if she was still staying with us, and I was like a 24/7 caregiver. But I don't, you know, I might stop in and see her once a week”—Caregiver 15
	Maintaining physical presence at hospital	*Maintaining active presence at hospital*: “I mean, essentially, I was in the hospital eight hours a day for, you know, two weeks.”—Caregiver 10
		Versus *less capacity to be present*: “I didn't spend a whole lot of time with him when he was in the hospital this time because … I had to get back to the office.”—Caregiver 9
Priorities for care	Curative versus comfort‐focused preferences	*Prioritizing work‐up and treatment for medical issues*: “You know, let's report this, so that, you know, it can be checked out. You don't want to jump to, you know, treating a behavior until you know all of the medical pieces are taken care.”—Caregiver 16
		Versus *a more maintenance‐focused approach*: “I think sometimes playing around with somebody's things when they're at the age that she's at is probably not always the best idea.”—Caregiver 6
Delirium knowledge	Familiarity with delirium and DSD	*Lack of familiarity with term*: “Delirium, yes. I ain't never really heard of that.”—Caregiver 20
		*Overlap with dementia*: “I don't really know how that [delirium] differs from regular dementia”—Caregiver 23
		*Knowledge from healthcare experiences*: “And so then she [geriatrician] explained to me about, you know, how this often happens with not just people with cognitive issues, but with patients who are in the hospital for a long time.”—Caregiver 17
Distress from delirium	Caregivers worry about impacts of DSD	*Impact of symptoms on caregiver*: “It was really very overwhelming, you know.”—Caregiver 18
		Versus *less concern*: “It's not abnormal for somebody 97 years old to be confused, and especially when there are changes.”—Caregiver 13
		*Impact of symptoms on patient*: “I think it's awful frightening for him.”—Caregiver 21

^a^ There was observed variation across all factors.

### 3.1 Phases of Navigating Acute Cognitive Changes

Caregivers to patients with a history of delirium (*N* = 19) described navigating acute cognitive changes through three interconnected phases: (1) recognizing acute cognitive changes, (2) interpreting their meaning and potential causes, and (3) deciding whether and how to act (Figure 1, Table 2).

#### 3.1.1 Recognizing Changes

The first phase involved the caregivers recognizing acute cognitive changes when they initially occurred. Notably, caregivers had limited awareness of the term “delirium” but readily described recognizing acute cognitive changes during illness. For example, Caregiver 1 said the term delirium was “new for me” but elaborated that “I'm very familiar with what can happen when my mom gets the UTIs, because it can affect how they think.” Changes were often recognized early, commonly before presentation to the emergency department.

Some caregivers recognized the patient experiencing changes to baseline alertness, behavior, or routines, noticing that the patient was “not themselves” or “not in their right mind.” Others identified that the patient seemed to be experiencing more confusion, hallucinations, or other behavioral or cognitive symptoms: “I find that when she has delirium, she's much more aggressive” (Caregiver 6). Caregivers reported that the fluctuating symptoms inherent to delirium could complicate recognition of and communication about symptoms. Caregiver 10 observed it was “very difficult to communicate… the sort of whole picture of things, you know, if you've got them looking great… one visit and looking awful the next.” Some caregivers relayed difficulty differentiating acute cognitive changes from cognitive impairment related to underlying dementia: “I don't know … if it's delirium or if it's a progression of his dementia” (Caregiver 9).

#### 3.1.2 Interpreting Changes

After recognizing acute cognitive changes, caregivers interpreted symptoms, considering possible causes and making sense of symptoms based on prior experiences. Some caregivers identified singular precipitants such as illness or anesthesia as the cause of symptoms. Other caregivers linked symptoms to more global disruption of familiar routines and environments: “Once we changed, you know, locations again, she just can't quite understand where she is.” (Caregiver 13). Caregivers also interpreted symptoms in the context of dementia, with some observing that baseline dementia symptoms worsened during hospitalization: “She repeats herself at home, but she tends to do it a bit more in hospital stays” (Caregiver 8).

Caregivers also interpreted the importance of addressing acute cognitive changes. Those who described the potential impacts (e.g., long‐term impacts on cognition) and modifiability of symptoms acknowledged the importance of addressing these changes to prevent further decline. Other caregivers interpreted changes in cognition as more normative and less important to address given their views on dementia or aging: “It is kind of a normal, you know, I mean, he does have dementia” (Caregiver 18).

#### 3.1.3 Deciding How to Act

Finally, caregivers decided how to act in response to acute cognitive changes, commonly seeking medical care, voicing concerns, sharing knowledge, and advocating for avoidance of perceived precipitants. Caregivers commonly described trying to relate patients' cognitive baseline and history to clinicians to convey that acute changes were distinct from the underlying dementia, informed by their interpretation of symptoms. Caregiver 6 recalled, “We try to explain like what her baseline is …. because sometimes some of her symptoms … is just kind of chalked off to dementia.” In another example, Caregiver 10 described advocating for avoidance of a perceived precipitant: “I finally was able to go the [emergency room] with her … and say, please, you know, no fentanyl.”

Caregivers also described how their earlier recognition and interpretation of acute cognitive changes informed whether and how they decided to act. When caregivers interpreted symptoms as a potential sign of acute illness, they often sought medical care. Some caregivers described acting due to concern about underlying medical causes, but not necessarily delirium itself, such as Caregiver 18: “I had seen him go through this once before when we actually had COVID … so I called the ambulance to come and take him in.” Several caregivers who were not present during the initial onset of symptoms (e.g., those living apart from the care recipient) were prompted to take action by others, including nursing home staff, but relayed symptoms to hospital clinicians.

### 3.2 Factors Shaping Caregiver Involvement in DSD Care

Caregivers described individual factors that shaped both how they navigated acute cognitive changes and their preferences for involvement in DSD care, including preferences for DSD education (Figure 1, Table 3). Specific factors shaping involvement varied by caregiver and included caregivers' views of their role and caregiving context, priorities for care, delirium knowledge, and distress from delirium.

#### 3.2.1 Caregiver's View of Their Role and Caregiving Context

Caregivers described distinct perceptions of their role—including the extent to which they viewed themselves as an advocate, proactive planner, and/or expert—that shaped how they navigated acute cognitive changes and their preferences for involvement in DSD care. Illustrating advocacy, Caregiver 1 observed that “I've become very aware that I need to make sure advocate for her on what I know, versus what the healthcare system knows” and subsequently engaged with the clinical team to ensure his mother received a work‐up for infection when she developed acute cognitive changes. Several caregivers described wanting to be prepared for future health issues in their caregiving roles and felt that proactive, anticipatory education about DSD would be helpful. For example, Caregiver 17 said “It's scary, and I think debriefing at the hospital, before they send him home to family, that would be excellent.” In contrast, other caregivers expressed that they would want education only as necessary and in the moment.

Contextual factors (such as caregivers' living arrangement with the patient) shaped caregivers' perceptions of their role in offering expertise to the clinical team regarding the patient's baseline cognition and/or detecting acute cognitive changes. Many caregivers felt that they had unique expertise to offer and wanted to be contacted frequently related to DSD care: “It's made me feel better to chat about like her baseline and give them some idea of like what is normal for her… I would much rather be called more often and asked more questions” (Caregiver 8). In contrast, some caregivers, particularly those living apart from the patient, conveyed that others (e.g., facility staff) may be more likely to detect changes: “So since he lives in like an assisted living, they are like the ones to like take care of him on a day‐to‐day basis and see any changes” (Caregiver 23).

Similarly, caregivers also noted differing capacity to be physically present at the hospital due to geographic factors or other responsibilities, which influenced their actions and involvement in care. For example, Caregiver 17 recalled that she was unable to accompany her spouse to the emergency department and the clinical team relied on the patient to provide a history because, “I had to take care of the dog and everybody at home and get everything locked up before I could leave.”

#### 3.2.2 Priorities for Care

Caregivers conveyed differing priorities and expectations for the patient's care that informed their actions and involvement. Several caregivers relayed that medical issues besides delirium were the focus of hospitalization, and that cognitive changes were not discussed extensively with hospital clinicians. For example, Caregiver 14 recalled that the clinicians were “more concerned with her actual health because they found a little pneumonia … I don't know if they talked about [cognitive changes] a lot.” However, other caregivers described seeking emergency care for acute changes in cognition as the primary concern, while simultaneously wanting to work up and treat any time‐sensitive medical issues. Others described more comfort‐focused priorities, relaying preferences towards avoidance of DSD precipitants and prognostic counseling to inform care planning: “[the doctor] basically told me that there are times when the symptoms won't get any better, and I thought that was the most truthful and important thing that I'd heard … because it helped determine the course of his care” (Caregiver 19).

#### 3.2.3 Delirium Knowledge

Caregivers' pre‐existing knowledge about delirium also shaped their recognition and interpretation of acute cognitive changes. Most caregivers described having limited familiarity with the condition or a lack of clarity about how delirium differs from dementia, potentially hindering their ability to recognize and interpret changes as DSD: “He probably was, yeah, experiencing some delirium, now that I know what it is” (Caregiver 9). Some felt that DSD education and a greater familiarity with the term “delirium” could facilitate caregivers recognizing symptoms; for example, Caregiver 1 felt that this knowledge could help him communicate his mother's history with delirium to clinicians: “Just to make sure that we mention it…throw the other word in there now.” Yet, not all caregivers felt that education would be helpful in identifying or responding to symptoms. Caregiver 7 explained that the overlap between delirium and dementia is “just like a wishy‐washy nothingness land that, I don't know how you'd distinguish one from the other.” When asked about whether any counseling he received was helpful, he observed “if [clinicians] did talk about [DSD], I was probably more dismissive of it because … I'm just kind of used to him being this way.”

#### 3.2.4 Distress From Delirium

The degree to which caregivers were concerned or alarmed by acute changes in cognition also shaped engagement preferences. For example, Caregiver 18 described high distress witnessing symptoms and expressed a desire for reassurance and emotional support: “I think I was looking for … maybe sympathy … and they always have been good about saying, well, now this often happens after surgery.” Others felt that education would be helpful for coping, such as Caregiver 8: “I feel like if she's experiencing or like showing these symptoms in the emergency room, like I think I would want [education] then almost as like a life raft or something to hold onto.” Conversely, other caregivers described less distress and did not perceive the need for improved caregiver support as a part of DSD care: “I mean, I don't know if there was a better way to approach that or not. I was fine with it” (Caregiver 15).

## 4 Discussion

Dementia caregivers described navigating acute cognitive changes during illness through three phases: recognizing changes, interpreting their significance, and decision‐making. There was substantial variation in how caregivers navigated these symptoms and their preferences for DSD care, including whether they believed involvement in DSD care was a high priority. These findings support flexible, caregiver‐centered intervention strategies for DSD that recognize variability in caregiver roles, needs, and readiness for engagement [28].

To optimize effectiveness, caregiver interventions for DSD should consider how caregivers interpret and respond to symptoms and incorporate caregiver expertise [12, 29]. Our work offers insights into additional considerations for future interventions. While caregivers frequently recognized acute cognitive changes, these symptoms were rarely conceptualized or described as delirium by caregivers, underscoring the need for clearer communication and education about DSD as a distinct and clinically significant syndrome. Delirium is described with numerous different terms, and more consistent terminology may help improve caregiver recognition of and understanding about this condition, including how delirium differs from cognitive fluctuations that occur as part of dementia [30, 31]. Caregivers may also observe acute cognitive changes during illness that do not meet diagnostic criteria for delirium; guidance on how clinicians should counsel in these situations may be beneficial.

Interventions that situate DSD in isolated care settings (e.g., hospital wards) may not match caregivers' lived experience given that many caregivers had already recognized, interpreted, and taken action in response to acute cognitive changes prior to hospital admission. There has been limited work examining community‐based interventions for delirium, which may provide more upstream education prior to hospitalization [12]. Building upon work by Shrestha et al., our findings suggest there is an opportunity to improve caregiver support regarding delirium recognition, interpretation, and management during pre‐ and post‐hospitalization care transitions [32].

Furthermore, hospital‐based interventions for DSD should account for the range of caregiver roles and contexts routinely encountered in acute care, as reflected in the different living arrangements and relationships in our sample. For example, hospital teams could ask about a caregiver's role, availability, and preferred level of involvement at admission to guide communication and engagement. Caregivers who are less available during hospitalization may benefit from simple, reliable communication approaches (e.g., scheduled updates or designed points of contact), whereas those who want to be more involved could help with non‐pharmacologic delirium prevention or contribute to structured assessments (e.g., FAM‐CAM) [33, 34]. Caregivers who live with the patient may be especially well positioned to share detailed knowledge of baseline cognition and identify acute changes in real time, whereas caregivers of patients residing in facilities may benefit from communication approaches that help bridge information across settings, such as personal passports that communicate information about patients' baseline cognition and support needs during care transitions [35].

While prior qualitative studies have found that dementia caregivers experience high distress witnessing DSD symptoms [29, 32], we observed substantial variability. One potential explanation for this difference is that distress may be shaped by patient and caregiver contextual factors, such as competing responsibilities and living arrangements. For example, Julian and Shrestha et al. exclusively interviewed caregivers to community‐dwelling adults with dementia, whereas our work also included caregivers to individuals residing at nursing facilities who may experience less distress [32]. Some caregivers experience high distress from DSD, and future work should examine how to effectively identify caregivers who would benefit from additional support [17, 36]. Additionally, dementia caregivers have other salient concerns beyond delirium that contribute to strain during hospitalization; these needs should be considered when implementing DSD‐specific interventions to not exacerbate burden [37]. Approaches that assess the individual caregiver's need and willingness to engage in an intervention over time, such as motivational interviewing, may be important for DSD interventions [38].

Our findings also reveal a tension between some caregivers' desire for reassurance and a potentially normative view of DSD symptoms and the poor outcomes associated with delirium [3, 5, 39]. Reassurance may reduce caregiver distress, but this could potentially downplay the clinical significance of DSD and lead to inappropriate caregiver expectations about recovery. Future work should attempt to clarify how to best navigate these tensions, potentially drawing upon frameworks such as serious illness conversations that recognize the need to balance patients' and caregivers' desire to be hopeful with prognostic awareness [40]. It is also possible that caregiver perceptions reflect that hospital clinicians may normalize delirium, and future efforts may need to address clinician attitudes and understanding of DSD in parallel [16, 41, 42].

Several limitations are present in our work. Participants were highly educated, and half reported professional healthcare experience. These characteristics may have facilitated caregiver advocacy, familiarity navigating the healthcare system, and recognition of clinical changes [43, 44]. Caregivers living in under‐resourced contexts or with less familiarity with healthcare systems may experience different barriers to engagement, potentially limiting transferability [43, 44, 45]. We did not restrict participation to primary caregivers; as a result, some participants may have had less comprehensive knowledge of the patient's baseline cognition, which could have influenced how they recognized and interpreted acute cognitive changes. Furthermore, our recruitment approach may have preferentially included caregivers who were more engaged and available, potentially underrepresenting perspectives of less‐involved or harder‐to‐reach caregivers. We determined the presence of delirium by chart review; reference standard ratings of the patient were not feasible for the present study. Interviews were conducted during hospitalization or within 1 week of discharge, and timing may have influenced responses, with interviews during hospitalization reflecting more immediate, experiential preferences and those after discharge allowing for greater reflection. The degree of variation in our data—across experiences with DSD, caregiving roles, living arrangements, and factors influencing preferences for involvement in DSD care—was analytically complex and merits further research to examine how these factors influence caregiver engagement longitudinally.

## 5 Conclusion

Our study findings underscore the complexity of caregiver experiences with acute cognitive changes during illness and preferences for involvement in DSD care. These findings highlight the need for more nuanced approaches to DSD intervention design that account for variability in caregiver roles, needs, and readiness for engagement.

## Author Contributions

Study concept and design: Blair P. Golden and Andrea Gilmore‐Bykovskyi. Data collection: Blair P. Golden and Brooke Johnson. Data analysis and interpretation: All authors. Manuscript preparation: All authors. Final approval of manuscript: All authors.

## Funding

This work is supported by the National Institute on Aging under Award Number K23AG081458 (PI Golden). Dr. Sharon K. Inouye's time is covered by National Institute on Aging Award Number R33AG071744 (PI Inouye). The content is solely the responsibility of the authors and does not necessarily represent the official views of the National Institutes of Health.

## Disclosure

The sponsor played no role in the design, methods, subject recruitment, data collection, analysis, or preparation of this paper.

## Conflicts of Interest

Dr. Sharon K. Inouye is the Editor in Chief of JAMA Internal Medicine, and holds the Milton and Shirley F. Levy Family Chair at Hebrew SeniorLife/Harvard Medical School.

## Supporting information


**Text S1:** Interview guide.
