# Supplementary material for: Dementia Caregivers' Preferences for Involvement in Delirium Care During Hospitalization
Source: J Am Geriatr Soc. 2026 Aug 3;74(10):2908–18. doi: 10.1111/jgs.70602 (PMC13523024; doi:10.1111/jgs.70602)
Supplement: Supplementary file 1 [file JGS-74-2908-s001.pdf]

Supplementary Text S1. Interview Guide

Section 1: General experience

1. Can you tell us a little bit about your relationship with the person that is hospitalized?
2. Can you tell me a little bit about what brought you all to the hospital?
3. Tell me a bit more about how the hospitalization has gone so far

Section 2: Prior experiences with delirium

1. What (if any) changes have you noticed in [the patient's] cognition since they have been in the hospital?
    - Did you communicate these differences to the clinical team? Why or why not?
    - How did these changes affect you?
2. Have you been told by a clinician (such as a nurse or doctor) that [the patient] is experiencing delirium?
    - If so, can you tell us about that experience?
    - What advice would you give to the hospital team about how to support caregivers with delirium?

Section 3: Delirium information needs

1. Have you ever heard about the condition of delirium before this hospitalization? If so, tell us about how you learned about it.
2. Do you think caregivers would benefit from more education about delirium? Why or why not?
3. What information or education about delirium would be most helpful to caregivers?
4. Are there any specific concerns or misconceptions that caregivers might have about delirium?

Section 4: Collaboration with hospital care teams

1. Some research has looked at having caregivers use tools to help determine if patients are experiencing delirium. Would you be open to helping identify delirium? Why or why not?
2. Do you have other ideas on how hospital teams could better identify delirium in patients with dementia?
3. Have any members of the clinical team (like a doctor or nurse) talked about things that increase the chances of delirium or ways to prevent delirium?
4. Have you previously tried anything to prevent delirium? What did you do?
5. Do you have other ideas on how hospital teams could better prevent delirium in patients with dementia?

Section 5: Conclusion

1. Before we conclude the interview, is there anything else you'd like to share about your experiences with the hospital?
